# Editorial: Innovation in tackling the global challenge of eradicating antibiotic-resistant microorganisms

**DOI:** 10.3389/fmicb.2025.1774105

**Published:** 2026-01-12

**Authors:** Israel Nissan, Avi Peretz

**Affiliations:** 1 Department of Avian Diseases, Kimron Veterinary Institute, Beit Dagan, Israel; 2 Clinical Microbiology Laboratory, Tzafon Medical Center, Poriya, Tiberias, Affiliated with Azrieli Faculty of Medicine, Bar Ilan University, Safed, Israel

**Keywords:** AMR, bacteriophage, genomic epidemiology, metagenomics, NGS, One Health, repurposed therapeutics, stewardship

Antimicrobial resistance (AMR) is no longer a distant risk; it is a daily constraint on routine clinical care, animal and plant health and the safety of food systems. The World Health Organization ranked AMR among the top global public health and development threats, with an estimated 1.27 million deaths directly attributable to bacterial AMR and 4.95 million AMR-associated deaths in 2019. These threats do not distribute evenly due to gaps in prevention infrastructure, diagnostics and access to effective therapy, resulting in heaviest burden on low-resource settings. In contrast, resistant pathogens and mobile resistance elements ignore borders and travel with patients, animals, food, water and trade.

In parallel, antibiotic innovation has been slow, with only 12 new antibacterial drugs approved between 2017 and 2021, most belonging to existing classes where resistance mechanisms are already established. This tension between rising resistance and limited therapeutic novelty, is pushing the field toward a dual mandate, namely, improving stewardship, prevention and diagnostics, while expanding the therapeutic and surveillance toolbox with alternatives to classic small-molecule antibiotics, new targets and One Health approaches that address reservoirs beyond the hospital.

This Research Topic, “*Innovation in tackling the global challenge of eradicating antibiotic-resistant microorganisms*,” presents 15 papers that reflect that broadened demands of innovation. The contributions span bacterial and fungal pathogens, hospitals, communities, soils and crops, with methods ranging from bacteriophage therapeutics and phage-derived enzymes to hybrid genome assembly, metagenomics, microbiome analytics and implementation of focused stewardship research. Together, they show that progress against AMR will require a combination of complementary tools, validated under real-world biological and operational constraints.

## Bacteriophages and phage-derived antibacterials: precision that faces biofilm reality

Bacteriophages are often framed as “precision antibacterials,” with performance depending on host range, bacterial physiology, clinical workflow and biofilm architecture. Using isothermal microcalorimetry, Lafranca et al. quantify the efficacy of a commercial phage cocktail against skin pathogens in planktonic culture and both thin and thick biofilms, and found that biofilm thickness and maturity can limit phage impact. They also noted that chronic wound settings may require biofilm disruption (e.g., mechanical debridement) to create conditions conducive to phage activity.

Two additional phage studies focus on the hypervirulent and multidrug-resistant *Klebsiella pneumoniae*. Peng et al. report the isolation, characterization and genomic analysis of a novel lytic phage (vB_Kp_XP4) targeting *K. pneumoniae*, and demonstrate that genomic inspection can support safer and more rational phage selection for therapeutic development. At the population level, Tellez-Carrasquilla et al. combine lytic phages with high activity against high-risk, globally disseminated *K. pneumoniae* clones (CG258 and ST307), and emphasize the essentiality of cocktail design and performance testing when lineages co-circulate and diversify.

In a plant-health context, Liu L. et al. investigate bacteriophage LDT325 as a biocontrol strategy against *Pseudomonas syringae*-associated bud blight in tea (*Camellia sinensis*). Their work links phage treatment to improved antioxidant defenses and enhanced disease tolerance, which can indirectly reduce antimicrobial use, selection and dissemination across agricultural and environmental interfaces.

Finally, the Topic includes a forward-looking perspective on “enzybiotics”—phage-derived lytic enzymes that may offer controllable, protein-based antibacterials. Bałdysz et al. argue that enzybiotic bioinformatics must mature to include better annotation standards, curated datasets and benchmarking that connects *in silico* predictions to measurable antimicrobial activity.

## Genomic epidemiology and resistome intelligence: tracking genes, plasmids, and transmission

Several papers in this Research Topic sharpen surveillance scope and capacities by addressing elements that traverse wards, institutions and bacterial lineages. Piispa et al. use hybrid genome assembly to resolve plasmids carrying carbapenemase genes across Enterobacterales isolated from patients and from multiple Finnish healthcare environments. Two complementary clinical datasets illustrate implications of local epidemiology on day-to-day decision-making. Liu Z. et al. analyze the distribution and resistance profiles of bacteria isolated from ICU blood cultures over several years, and provide evidence that can refine empiric therapy and stewardship in high-risk settings. Qiu et al. characterize carbapenem resistance mechanisms in clinical *K. pneumoniae* isolates and examine genotypic-phenotypic correlations and transmission patterns. Together, these studies reinforce the need for strict surveillance of “units of spread,” which can be a clone, lineage, plasmid, or even an ecological reservoir.

## One Health perspectives: plants, soils, and livestock as AMR arenas

Using metagenomics, Yu et al. profile soil microbial communities, antibiotic resistance genes and virulence factors in tea plantation soils after two decades of conventional vs. organic management. Their work shows how agricultural practice and environmental conditions can shape the background resistome, i.e., the genetic “starting point” from which resistance determinants may persist or spread.

Wagner et al. address reliance on relative abundances in standard 16S rRNA amplicon sequencing, which can be misleading when total microbial loads shift and is biased due to variable 16S copy numbers. By incorporating absolute abundance calculations, they show a substantially different inferred impact, both in magnitude and statistical significance, of antibiotic treatment on the fecal microbiota of young pigs. This methodological advance can reshape how we interpret intervention in human and veterinary medicine and, consequently, how we assess selection pressure and downstream AMR risk.

## Stewardship and implementation: making innovation durable

Using an interrupted time-series approach, Abdelsalam-Elshenawy et al. examine how seasonal variation and the COVID-19 pandemic influenced antimicrobial stewardship activities and antibiotic prescribing for respiratory tract infections in a UK secondary care setting. Their results demonstrate that external shocks and seasonal pressures can rapidly shift prescribing behavior and therefore, stewardship programs must be designed to remain effective when routines are disrupted.

## Novel or repurposed therapeutics and targets

Aloriby et al. evaluate the efficacy of *Olea europaea* and *Ficus carica* leaf extracts against multidrug-resistant pathogens through an integrated pipeline combining *in vitro* testing, *in vivo* toxicity assessment and *in silico* modeling. Their workflow illustrates a disciplined path for prioritizing plant-derived antimicrobial candidates.

Shaalan et al. examine three urease inhibitors (acetohydroxamic acid, ebselen and baicalin) and their effects on *Helicobacter pylori* viability, urease activity and urease gene expression. Their insights can be applied to weaken colonization-critical functions which could complement antibiotic regimens and counteract the rising resistance-driven *H. pylori* treatment failure.

Wang et al. combine virtual screening targeting the transcriptional regulator AbaA, with experimental validation and transcriptomic analysis to identify small-molecule candidates active against *Sporothrix globosa* and to illuminate underlying response pathways. In *Candida glabrata*, Zheng et al. uncover a connection between tunicamycin-induced respiratory deficiency and reduced fluconazole tolerance, emphasizing that tolerance phenotypes can be governed by cellular physiology and may represent actionable vulnerabilities.

## Outlook: integration is the strategy

These 15 papers do not claim a single solution for AMR. Instead, they offer a portfolio of approaches that resolve the vehicles of resistance, provide metagenomic views of environmental reservoirs, improve microbiome analytics, develop novel or repurpose molecules in both bacteriology and mycology and research system-level behavior.

The unifying message is both sobering and hopeful. No “silver bullet” will eradicate antibiotic-resistant microorganisms. Durable progress will arise from integration of new therapeutics with improved diagnostics, faster genomic surveillance and stewardship programs resilient to real-world pressures. By building bridges between bench and bedside, farm and clinic, genome and guidelines, the innovations showcased here can transition from promising studies to agents with sustained impact on the fight against antibiotic-resistant microorganisms.

